# Age-Dependent Recombination Rates in Human Pedigrees

**DOI:** 10.1371/journal.pgen.1002251

**Published:** 2011-09-01

**Authors:** Julie Hussin, Marie-Hélène Roy-Gagnon, Roxanne Gendron, Gregor Andelfinger, Philip Awadalla

**Affiliations:** 1 Department of Biochemistry, Faculty of Medicine, University of Montreal, Montreal, Canada; 2 Sainte-Justine Hospital Research Centre, Montreal, Canada; 3 Department of Social and Preventive Medicine, Faculty of Medicine, University of Montreal, Montreal, Canada; 4 Department of Pediatrics, Faculty of Medicine, University of Montreal, Montreal, Canada; University of Oxford, United Kingdom

## Abstract

In humans, chromosome-number abnormalities have been associated with altered recombination and increased maternal age. Therefore, age-related effects on recombination are of major importance, especially in relation to the mechanisms involved in human trisomies. Here, we examine the relationship between maternal age and recombination rate in humans. We localized crossovers at high resolution by using over 600,000 markers genotyped in a panel of 69 French-Canadian pedigrees, revealing recombination events in 195 maternal meioses. Overall, we observed the general patterns of variation in fine-scale recombination rates previously reported in humans. However, we make the first observation of a significant decrease in recombination rates with advancing maternal age in humans, likely driven by chromosome-specific effects. The effect appears to be localized in the middle section of chromosomal arms and near subtelomeric regions. We postulate that, for some chromosomes, protection against non-disjunction provided by recombination becomes less efficient with advancing maternal age, which can be partly responsible for the higher rates of aneuploidy in older women. We propose a model that reconciles our findings with reported associations between maternal age and recombination in cases of trisomies.

## Author Summary

Aging is a genetically and environmentally modulated process. One particular manifestation of aging in humans is the age-related changes that affect the female reproductive system. It is well established that chromosome-number abnormalities in offspring occur more frequently as maternal age advances, but the meiotic mechanisms involved remain unclear. Meiotic recombination has been associated with maternal age in different species but contrasting effects of maternal age on recombination rates have been reported among mammals. In this study, we found a decrease of recombination rates with increasing maternal age in a French-Canadian cohort, with the most pronounced decline possibly occurring before 32 years of age. We observed chromosome-specific age effects, and in older women recombination frequencies are notably reduced in the middle portion of chromosomal arms and near subtelomeric regions. No paternal age effect on recombination was found, highlighting differences in patterns of variation among sexes. Many studies have shown significant inter-individual variation in genome-wide recombination rates, and our results points to an additional, intra-individual source of variation in recombination rates among transmissions from the same mother.

## Introduction

Meiotic recombination is crucial in both driving the evolution of genomes and ensuring faithful segregation of pairs of homologous chromosomes during gametogenesis. The initiation of genetic recombination during the first meiotic prophase enables homologous chromosomes to orient properly on the spindle and helps form physical connections between chromosomes [1]. This process results in strand crossovers and further leads to zygotes harboring new combinations of parental genetic material. Every descendant is therefore provided with a unique mosaic of both pairs of parental chromosomes.

In most mammals, including humans, there are important sex-differences in recombination rates and patterns [2]. First, the distribution of crossovers along the genome differs between sexes, tending to be lower at the telomeres in females relative to males [3]. Second, the average size of the genetic map for females is 1.6 times longer than that for males [4], [5]. Third, 15% of female and male hotspots are sex-specific [6]. Evidence indicates that these differences result from sexual dimorphism in the regulation of the meiotic process [7]–[9], but high levels of heterogeneity in recombination rate is also observed within the same sex.

Pedigree studies have identified extensive variation in rates among females [3], [4] and more recent studies reported significant variation in both female and male crossover rates [6], [9]–[11]. In addition to interindividual variation, the number of crossovers among different gametes of an individual has been reported to vary [12], [13]. However, variation in gamete recombination does not necessarily translate into variation in offspring recombination, since only a small subset of gamete variation may be consistent with live-born offspring. For example, more than 20% of oocytes exhibit an abnormal number of chromosomes, and yet very few aneuploid embryos are viable [7]. Since reduction or failure of meiotic recombination is associated with improper disjunction of chromosomes, leading to genetically unbalanced gametes, high rates of recombination protect oocytes from non-disjunction events [1], [14], and oocytes with many crossovers are likely to result in a live embryo. Conversely, oocytes exhibiting too few crossovers are particularly prone to aneuploidy.

The most important factor linked to chromosomal aneuploidy in women is advancing maternal age [7]. Since very little is known about age-related causes of non-disjunction, it remains important to establish associations between patterns of recombination and maternal age in normal meioses. Although recombination is initiated during fetal development in mammal females, age can still influence recombination. In mice, it has been demonstrated that oocytes do not exit the mitotic phase of oogenesis all at once, but rather in successive waves [15]. Furthermore, oocytes ovulate in the same order in which they entered meiosis [15]. This ‘production line’ model thus suggests that eggs ovulated late in life are the result of more premeiotic mitotic divisions.

Contradictory observations for relationships between maternal age and recombination rates have been reported in mammals, with studies reporting weak increases with age of recombination count estimates in humans [11], [16] whereas in mice and hamsters decreases in frequency of crossovers with age were reported [17], [18]. To further investigate the maternal age effect on recombination in humans, we densely genotyped individuals from 68 French-Canadian families in Quebec and localized recombination events at high resolution using a previously described method [11]. We report a significant genome-wide decrease in recombination rate with advancing maternal age in humans and we compare our results with observations from similar studies. Chromosome-specific effects likely drive the observed reduction in recombination with age. Our observations are consistent with a proposed model in which protection against non-disjunction through recombination becomes less efficient with advancing maternal age in some chrosomosomes.

## Results

### Significant variation in fine-scale recombination patterns

Maternal recombination rates in meioses can be examined by inferring recombination events in viable offspring using dense genome-wide genotyping of pedigrees. To capture crossovers occurring during parental gametogenesis, a total of 478 individuals from 68 French-Canadian pedigrees were genotyped using the Affymetrix 6.0 1 M Chip. Over 650,000 SNPs were retained after stringent quality control, providing information on 195 maternal and paternal meioses. Following the procedure described by Coop and colleagues [11], we localized crossovers at high-resolution in 68 nuclear families with at least two children and examined variation in fine-scale recombination patterns among individuals. We observed an average of 41.7 (40.2–43.3 95%CI) and 27.7 (26.9–28.4 95%CI) recombination events among maternal and paternal transmissions, respectively, in close agreement with published estimates [4], [9]–[11], [19].

We confirmed the presence of significant variation for fine-scale patterns of recombination [9], [11], suggesting that we have sufficient power to detect fine-scale variation patterns among individuals in this cohort. In particular, we observed significant variation in recombination rates among males and females for individual chromosomes (Table S1), including chromosome 19 in males. Although recombination is positively correlated with gene density, chromosome 19 has been previously reported to be an outlier, as this chromosome has the lowest density of recombination hotspots [20] but the highest gene density [21]. It also carries the highest proportion of open chromatin [22]. We also evaluated the overlap between the recombination events inferred in our cohort and known population recombination hotspots inferred from HapMap3 CEPH haplotypes. To do so, we considered a subset of recombination events inferred to be less than 30 Kb apart. We found that 70% and 68% of maternal and paternal events, respectively, overlapped described recombination hotspots [20], whereas less than 35% overlap is expected if recombination events are randomly distributed across genomes. Overall, these results demonstrate that there is substantial heterogeneity in recombination counts among families, sexes and individuals.

### Genome-wide negative maternal age effect

**Figure 1:**
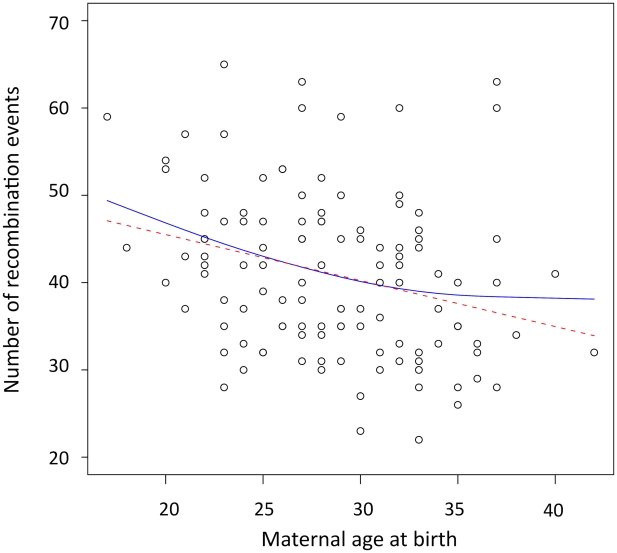
Scatterplot and fitted regression functions showing negative correlation between the maternal age at birth and the number of recombination events in offspring. The red dashed line is the linear regression (β = −0.49, *p* = 0.0017, r^2^ = 0.081). The solid blue line represents the result of the linear spline regression with knots at each distinct value of maternal age at birth and the smoothing parameter λ estimated by REML (λ = 22.29, *p* = 0.005, r^2^ = 0.091).

The number of observed crossovers in children of our cohort is negatively correlated with maternal age at time of birth (β = −0.49 crossovers/year, Pearson r = −0.28, *p* = 0.0017). This negative maternal age effect was determined using a linear mixed model that account for the effects of the mother on recombination rates. This effect remained significant after including the number of children of a mother as a covariate in the model (β_age_ = −0.44 crossovers/year, *p* = 0.007). We also used family-adjusted recombination counts and ages to evaluate if the age trend detected exists ‘within family’ (see Materials and Methods). Maternal age remained negatively correlated to the number of recombination events across transmissions within families (β = −0.42 crossovers/year, Pearson r = −0.25, *p* = 0.0047), ruling out the possibility that this pattern is due to variation in recombination rates among mothers. To determine the period of reproductive life in which the maternal age effect is strongest, we used a linear spline smoothing while specifying a random effects structure to account for the within-family correlations [23]. The fitted spline regression is displayed in Figure 1 along with the fit of the linear regression. The spline fit suggests that recombination counts decrease for all ages, with the greatest decline found among children born from mothers that are 32 years of age or younger. We note, however, that the spline fit is not a significant improvement relative to the linear fit (*p* = 0.0695).

Most double recombination events called within less than 1 Mb of each other were a result of genotyping errors (see Materials and Methods), nevertheless, including these events did not change the direction of the negative trend with age observed in females. Even when called double recombination events occurring within 2, 5, 10 and 20 Mb were excluded from the analyses, the significant negative correlation between recombination counts and maternal age remained (Table S2). All analyses were also performed for males, and no significant correlation was observed between paternal age and the number of paternal crossovers inferred (with family-adjusted values: β = −0.18, Pearson r = −0.15, *p* = 0.12) as previously reported [11].

### Evaluating maternal age effects along chromosomal arms

**Figure 2:**
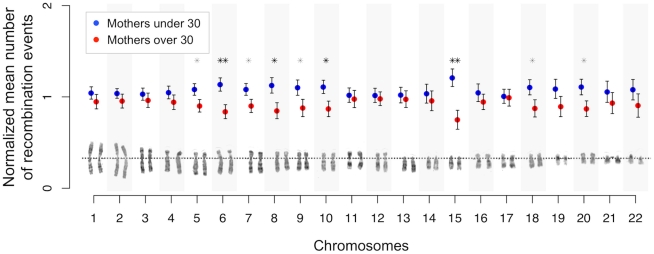
Chromosome-specific shifts in normalized means (and standard errors) of the number of maternal crossovers for mothers under and over 30 years of age. Position of centromere is shown for each chromosome (dotted line). Significance of the shift at the 5% (*) and 1% (**) levels is assessed by permutations.

We investigated whether the observed genome-wide negative correlation between maternal age and crossover counts is specific to certain chromosomes or genomic regions. For all chromosomes, the mean number of crossovers observed in mothers older than 30 years of age was less than for younger mothers, and significantly so (*p*<0.05) for chromosomes 5 to 10, 15, 18, and 20 (Figure 2 and Table S3). Putting aside these nine significant chromosomes, the observation that the remaining chromosomes all show reduced mean recombination rates in older mothers (*p* = 1.22·10^−4^) is a robust signal for a systematic negative effect. However, the negative correlation is no longer significant for these individually non-significant chromosomes grouped together (with family-adjusted values: β = −0.13, r = −0.14, *p* = 0.101), suggesting that the genome-wide effect detected is mainly driven by effects present on specific chromosomes. Also, the shift in mean between younger and older mothers seen for the above significant chromosomes is significantly greater than that for the remaining chromosomes (one-tailed *p* = 1.2·10^−3^). From simulations, we noted that no more than seven chromosomes would be expected to be significant if the genome-wide effect is shared uniformly across all chromosomes (see Materials and Methods). Therefore, our result of nine statistically significant chromosomes appears to be an outlier (one-tailed *p*<0.0002) where submetacentric chromosomes are overrepresented (7 out of 9, one tailed *p* = 0.043), suggesting that chromosomal arm size or structure are potential determinants.

**Figure 3:**
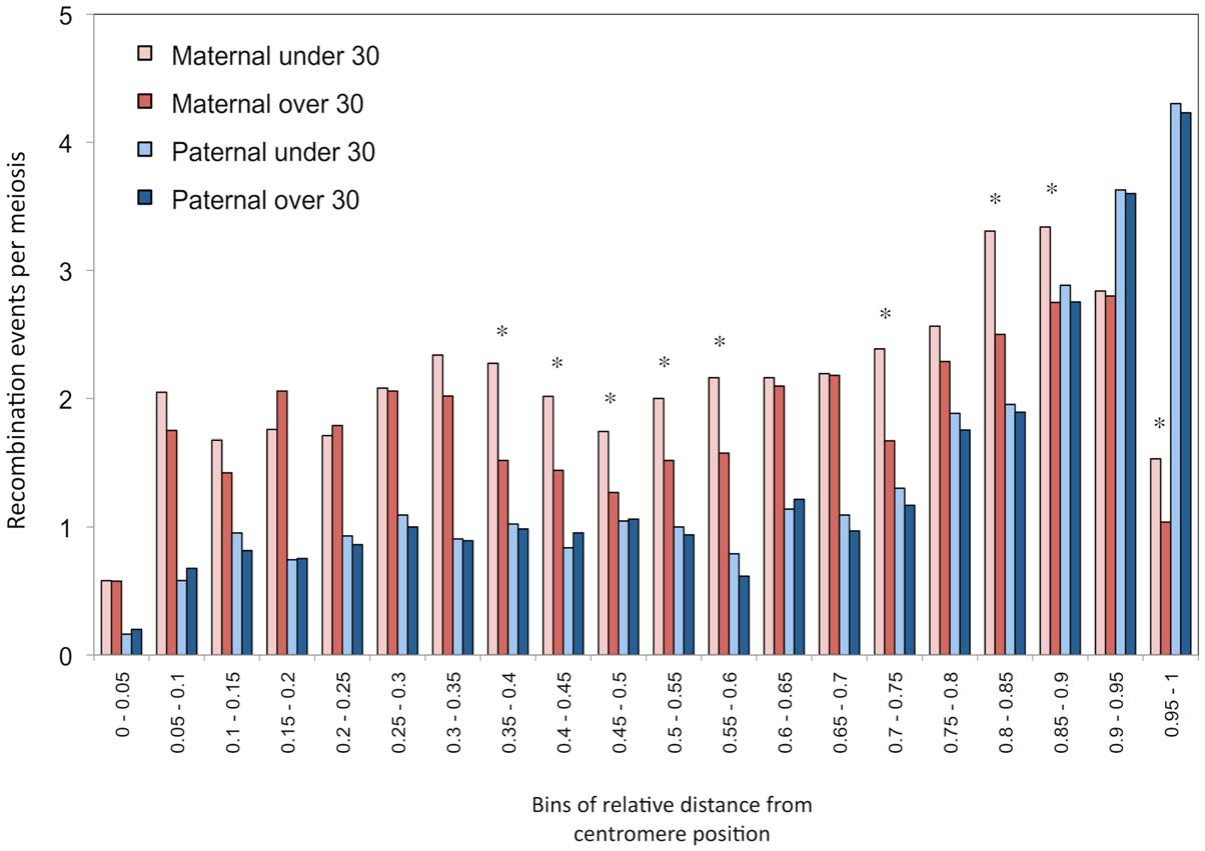
Distribution of recombination events along chromosomal arms. Histograms of mean number of events per transmission, grouped in 20 bins of relative distances from centromere (increments of 0.05 units). Paternal and maternal events are shown separately and transmissions are partitioned according to the age of the parent at birth. Parents of 30 years old are part of the over-30 groups. Significance of the shift at the 5% level (*) is assessed by permutations. All autosome arms are included.

We computed the distribution of maternal and paternal recombination events along chromosomal arms for parents under and over 30 years of age, independently (Figure 3). Male recombination rates increase as we approach telomeric ends of chromosomes, as seen in other studies [3], [4], [24]–[26], whereas female rates drop substantially at subtelomeric regions. The difference in recombination counts between mothers from the two age groups is clearly visible and no such pattern is seen in males. The statistical correlation between recombination counts and maternal age was evaluated with respect to relative location on chromosomal arms (Table S3). The decay in crossovers with maternal age appears to be localized in specific portions of chromosomal arms. More precisely, the reduction in recombination rates observed for older mothers in the middle section of chromosomal arms and near the subtelomeric regions is significantly greater than those in the other bins (one-tailed *p* = 0.0464).

Finally, we compared recombination hotspots locations between mothers younger and older than 30 years of age. A large proportion of events (70%) overlapped with previously identified population recombination hotspots in both age groups. Furthermore, no significant differences were found among younger and older mothers in the distribution of hotspots along chromosomal arms (Figure S1).

### Phenotypes show no association with maternal age and recombination

Among our study cohort, 40 children have left-sided congenital heart disease (LS-CHD), a cardiac malformation where there is substantial evidence for a genetic component [27]–[29]. We therefore tested for possible associations between the disease phenotype and both maternal age at birth and recombination rates, and found none. Moreover, the negative correlation between family-adjusted crossovers and maternal age remained significant when only unaffected children were considered (*p* = 0.0023). Five mothers had LS-CHD and were involved in 21 transmissions. Again, a significant negative correlation between family-adjusted values is observed when these transmissions were removed from the analysis (*p* = 0.0059). These results indicate that clinical phenotypes in a subset of our study cohort have little to no effect on our findings.

### Comparisons with previous studies in humans

Our main finding that the maternal age effect is negatively correlated with recombination rate is in sharp contrast with a previous finding in an Icelandic cohort [16] where a positive correlation between maternal age and recombination rates was observed. There are three main differences in design between the two studies. First, the Icelandic study has a much larger sample size, perhaps making feasible the detection of what is a very weak positive effect (β = 0.043 recombination events per year) that may not have been detectable in our study. Second, approximately 1000 microsatellite markers, of unknown location or distribution, along chromosomes were used to map recombination events. Third, maternal age at birth was approximated by rounding ages up to the nearest five years. Through simulations, we showed that the discrepancy in results between studies is unlikely to be due to sample size effects (Text S1). To evaluate to what extent the number of sampled markers and age approximations affect the power to detect an effect among the French-Canadians, we recreated these conditions with our dataset. When we grouped maternal ages the same way as in the Icelandic study and used 1000 randomly selected informative markers per mother, the original negative trend remained but the correlation was no longer significant at the 5% level. The mean number of crossovers across transmissions for younger mothers drops from 43.07 to 35.13. For older mothers, the mean drops from 38.04 to 31.62 crossovers per transmission, shifting the difference in means between younger and older mothers from 5.03 to 3.51 crossovers. Finally, our correlation remained significantly negative when we used only 100 000 SNPs, corresponding to 6000–7000 informative markers in our analysis.

Coop and colleagues also reported a positive effect observed among related Hutterites [11] and kindly provided us with recombination rates and parental age at birth in 52 of their nuclear families. Both marker density and the methodology used to infer recombination rates are similar in our studies. Using the data provided, we reproduced their finding and observed a significant positive correlation between recombination counts and maternal age using a linear mixed model (Figure S2a) and family-adjusted values (β = 0.22, Pearson r = 0.13, *p* = 0.034), however with an explained variance in recombination rate of less than 2%. Moreover, non-parametric tests showed no significant correlation (Spearman ρ = 0.10, *p* = 0.11). All results remain unchanged when only recombination events seen once in a family are kept in the analyses.

Among the Hutterites, the distribution of maternal and paternal recombination events along chromosomal arms is very similar to those observed in the French-Canadian cohort (Figure S2b), except that the age effect is barely visible. The positive effect does not seem to be specific to particular chromosomal regions, since the correlations were not significantly different between regions. When examining chromosome-specific age effects among the Hutterites, no significant increase was observed on any chromosome (Table S4). However, two chromosomes showed a significant reduction in the mean number of crossovers for mothers over 30 years of age: chromosomes 20 (*p* = 0.0354) and 22 (*p* = 0.0321) with a one-tailed probability of 0.0455 that at least two chromosomes exhibit such p-values by chance alone (see Materials and Methods).

In order to compare to data in the Icelandic study [16], where age data was binned in age categories of five years, we binned the French-Canadian and Hutterite data into similar age categories, for each cohort separately (Figure S3). We observed significant differences in recombination rates among categories (*p* = 5·10^−4^) in the French-Canadians, but not in the Hutterites (*p* = 0.091). It is worth noting that the average number of crossovers per transmission decreases between mothers aged 25 to 29 and those aged 30 to 34 at time of birth in the Hutterites and in the Icelanders (see Figure 1 in [16]), although the differences are not significant.

## Discussion

In this study, we examined age-related effects on recombination and observed a negative correlation between the number of maternal crossovers and the mother's age at the time of birth. The proportion of the total variance explained by the genome-wide correlation is significant, yet relatively small (8.1%). This observation is striking considering no strong effect is expected, because considerably reduced levels of recombination are associated with non-viable offspring. The maternal-age effect is pronounced in the middle and distal portions of chromosomal arms. The decrease in recombination might be more pronounced for mothers younger then 32 years of age, after which the rate of maternal non-disjunction is reported to accelerate [7].

The possibility that age might influence recombination rates has been examined in several organisms. An age-related decline in recombination has been demonstrated in plants and Drosophila [30], [31]. However, in the latter an increase at older age (>16 days) has been reported [31], [32]. In mammals, while maternal age has been associated with recombination rate in several studies, paternal age effects on recombination have not been demonstrated. This asymmetry may be explained by important differences in the time of entry, duration and outcome of meiotic processes between sexes [7], [8]. While male germ cells are produced continuously and progress from prophase I to the second meiotic division in several days, the life cycle of oocytes is longer and more complex, beginning during early fetal life [33]. After a period of mitotic proliferation, oocytes progress through prophase I and initiate genetic recombination, before entering an arrest phase. In humans, meiotic arrest can be maintained for decades, until the oocyte resumes the first meiotic division and proceeds to metaphase II, prior to ovulation. In each of these meiotic stages, errors affecting chromosome segregation may occur and become more frequent as women age [34]. Particularly, the physical manifestation of recombination has a critical role in tethering homologous chromosomes together during meiosis [1], and a significant reduction in recombination has been identified as a causal mechanism underlying non-disjunction of pairs of chromosomes [13]. If the association we observe in this study reflects reduced recombination in oocytes ovulated later in life, one might consider this reduction to be partially responsible for higher level of aneuploidies in older women.

While our results are in agreement with the effects reported in mammals, they directly contradict previous studies demonstrating recombination rates increase with maternal age in humans [2], [11], [16]. All other analyses we performed studying recombinational patterns among families, sexes and individuals corroborate results found in other cohorts. It is possible that fewer markers or possible misspecification of maternal ages at birth in the Icelandic cohrt contributed to differences in these studies. Furthermore, the effect of maternal age on recombination rate reported was very small [16] (0.043 crossovers per year). We showed that our effect, which is almost 10-times stronger in the opposite (negative) direction, would not have been detectable in our sample using a similar marker density and maternal age estimation [16]. Further analyses of the Icelandic cohort, using recently published data [6], may be informative.

The positive correlation in the Hutterite cohort is also very weak, albeit significant [11]. Our data and methods are very similar and are unlikey to be the cause of the discrepancy. There may be two remaining explanations that can account for the differences between the two studies: either the populations are intrinsically different or the studies are capturing different aspects of variation in female recombination rates. It is possible that the different trends observed in these studies reflect a variable age phenotype in females [9], [19]. In humans, genetic determinants could have evolved to counter an age-related reduction in recombination, as it is observed in rodents, leading either to the absence or a weak increase of recombination rates [16]. If these phenotypes coexist in human populations, one may observe increasing, decreasing or even U-shaped trends in any given population. Moreover, the selective pressure acting in humans is unlikely to act in rodents, who rarely exhibit age-related meiotic dysfunctions, allowing the negative maternal age effect to be observed consistently.

Because of the association between aneuploidy and recombination rates, women that do not harbor an « age-dependent reduction » recombination phenotype will tend to have more children later in life. This would lead to a potential slight increase in the mean number of recombinations for mothers having more children [11],[16]. We note that there are a greater proportion of larger families in both the Icelandic and the Hutterites cohorts compared to our cohort. If the risk of non-disjunction or other chromosomal anomalies increases with age, then only oocytes with more recombination will survive and be observed in larger families.

**Figure 4:**
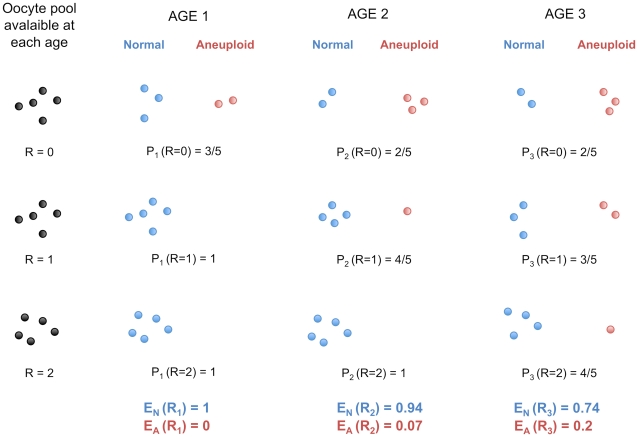
Protection against non-disjunction may be reduced as women ages. We propose that protection given by high recombination becomes less efficient with increasing maternal age. Here, we depict oocytes containing only one chromosome, with R recombination events. We suppose that, at each of the three arbitrary age periods (k = 1,2,3), the proportion of oocytes having R recombinations stays the same (i.e. it does not decrease or increase with age). During each age period, several oocytes enter their final stage of maturation and give properly disjoined gametes with one chromosome (Normal) or non-disjoined gametes with zero or two chromosomes (Aneuploid). P_k_ (R = *r*) is the probability of proper disjunction in an oocyte with *r* crossovers for the age period k. *E_N_* and *E_A_* are the mean numbers of recombination in properly disjoined and non-disjoined oocytes, respectively. Under this model, *E_N_* is expected to decrease with k whereas *E_A_* is expected to increase with k (see Text S1).

The age-effect observed among French-Canadians may also be a consequence, and not a cause, of the higher frequencies of non-disjunction with advancing maternal age. The patterns we observed for viable offspring do not necessarily reflect a decrease of recombination among oocytes in the female ovary, where all eggs might be recombining at the same rate. Rather, the number of crossovers sufficient for proper homologous segregation in young women may not be protective against non-disjunction in older women. More oocytes would give rise to aneuploid zygotes when ovulated later in life, a model consistent with the observations that increasing age of women increases the likelihood of trisomy. Under this hypothesis, one can show that the mean number of crossovers observed is expected to increase with maternal age in aneuploid conceptions and to decrease in normal, properly disjoined, fertilized eggs (Figure 4 and Text S1).

Trisomy studies provide evidence that recombination rates may increase with maternal age in aneuploid conceptions. Robinson and colleagues [35] studied non-disjunctions of chromosome 15 and reported that the mean maternal age at birth for cases harboring more than two crossovers is substantially higher than for cases with zero, one or two crossovers. This suggests a positive association between maternal age and recombination rate in aneuploid conceptions involving chromosome 15, and similar associations have been reported for chromosomes 18 and X [36], [37]. In trisomy 13, 16 and 21, however, no age-effect was reported [38]–[41] except for one study in trisomy 21 which found such an effect [42]. Interestingly, in the normal conceptions studied here, chromosomes 15 and 18 had a significant decrease in recombination with maternal age, whereas no significant effects were found for chromosomes 13, 16 and 21. Therefore, our model is consistent with significant association found for some chromosomes but not for others [7], [43]. Not all trisomies are affected by increasing maternal age equally and it seems unlikely that the same mechanisms apply to all aneuploid conceptions [44].

Altogether, these data highlight the fact that different chromosomes are subjected to distinct selective, mechanistic or structural constraints influencing recombination patterns over successive generations. This points to chromosome-specific effects that might be critical determinants of the complex relationship between maternal age and recombination in humans. Chromosome-specific effects may vary among populations depending on genetic differences in factors regulating the recombination machinery. Our results support this hypothesis, as a significant decay was found on nine chromosomes in our French-Canadian cohort and on two chromosomes in the Hutterites. The result for chromosome 20 was significant in both cohorts, but the overlap could be explained by chance alone.

Many factors could reduce the protection provided by recombination from meiotic breakdown, such as factors acting when meiosis resumes after arrest during the final stages of oocyte growth and maturation [45]–[47]. Furthermore, factors related to the functional significance of telomeres in meiotic recombination might be implicated. According to the telomere theory of reproductive aging in women [48], [49] shorter telomeres could be detrimental to segregation of chromosomes, especially for those with recombination event near subtelomeric regions [50], [51]. Moreover, the telomere length and rate of erosion might be associated with sex- and chromosome-specific genetic factors [52], [53] that vary among human populations or cohorts.

In conclusion, high-density genotyping of nuclear families enabled us to capture individual heterogeneity in recombination rates. The results described here are in favor of adaptative theories of sex-specific recombination rates [2] suggesting that increased rates in females may have evolved to compensate for improper chiasma formations later in life. The biological causes that underlie recombinational variation and sex-differences have been under investigation [9], [19], [54], [55], but the implications of variable rates for population genetic inferences and disease mapping remain unknown.

## Materials and Methods

### Ethics statement

The ethics committee of Sainte-Justine Hospital Research Center, University of Montreal, approved the study protocol and all participants gave their informed consent. The study was in accordance with the principles of the current version of the Declaration of Helsinki.

### Cohort description and genomic data

A French-Canadian cohort was recruited to discover genomic variants contributing to left-sided congenital heart disease (LS-CHD). The cohort is composed of 68 three-generational French Canadian pedigrees, together consisting of more than 700 individuals, including 242 individuals affected with LS-CHD. All participants underwent physical exams, ECG and echocardiography. A total of 478 individuals from 89 overlapping nuclear families were genotyped using the Affymetrix 6.0 platform. Further analysis of this cohort will be presented elsewhere.

We applied standard quality control SNP filters such as call rates (<95%), departures from Hardy-Weinberg (*p*<0.01), replicate concordance and Mendelian errors, resulting in a data set of 657,823 autosomal polymorphic SNPs. Genotypic data are available (Dataset S1).

### Algorithm to call recombination events

To localize crossover events in autosomes, we only considered the 69 nuclear families in the French–Canadian cohort that had at least two children. We used a previously described heuristic algorithm [11] that identifies parental informative markers and phases each child using sibling information. Three modifications to the procedure reported by Coop et al. [11] were made. First, in order to compare recombination rates among families, we evaluated the same SNPs in all families, removing 209,816 SNPs with missing data in at least one family (Dataset S1). Second, to filter out potential remaining genotyping errors, we discarded double recombinants over short intervals. We used a pre-treatment strategy to remove SNPs that result in an observed double recombinant, inferred within 1 Mb (∼1 cM, with genomic average of 1 cM/Mb) rather than discarding double recombinants occurring within five informative markers [11]. The majority of double recombinants removed were found in many individuals at the same positions and are therefore unlikely to be real double-crossover events. Third, the Coop et al. algorithm counts as recombination events the crossovers that are not unique in large families (with four children or more). This means that two offspring can have the same recombination event occurring between the same markers. For smaller families however, only events classified as unique would be captured. This leads to a downward bias in the total number of events detected in small families, relative to larger families [11]. Thus, in our analyses, we chose to only consider crossovers that are unique in both small and large families. Because this can lead to a downward bias in the number of crossovers for larger families, we partitioned large families into all possible combinations of families of three children (reduced families). For every child, we inferred the recombination counts for the reduced families that include this child, and computed the unbiased recombination counts, averaged over all reduced families. All the results presented in this study remained statistically significant when unbiased recombination counts were used.

To ensure that variation in call rate did not lead to miscalling of recombination events, we examined the correlation between genotype call rates and inferred recombination rates. The number of recombination events observed in a child is uncorrelated with the genotype call rate in this child (Spearman ρ = 0.098, *p* = 0.29 for maternal transmissions). The mean number of recombination events per mother is not correlated with the genotype call rate in the mother (Spearman ρ = −0.035, *p* = 0.71).

### Fine-scale recombination patterns among individuals

On average, 23,165 informative markers per transmission were used to infer recombination events in our cohort. To verify whether we had sufficient power to detect variation in fine-scale recombination patterns among individuals, we computed the average number of recombination events inferred among maternal and paternal transmissions. Confidence intervals were estimated by bootstrap. Following [11], we confirmed the presence of significant variation in the mean number of events genome-wide among females (*p* = 0.0032) and males (*p* = 0.0065) using ANOVA. We detected significant variation among individual chromosomes using a linear mixed model that corrects for genome-wide variation in recombination rates [11]. Significance was determined using a randomization procedure whereby children were randomly reassigned to parents without modifying family sizes. We assessed the congruence of Phase II Hapmap recombination hotspots [20] with events localized between informative markers less than 30 kb apart, because the location of these events is considered to be more accurate. The expected proportion of events overlapping a hotspot by chance has been computed as detailed by Coop and colleagues [11].

### Correlation between recombination and maternal age across transmissions

To study the correlation between recombination in offspring and maternal age at birth, we considered the 34 nuclear families with more than two genotyped children, because with only two children the number of events in each child cannot be determined. Following Kong and colleagues [16], we used a linear regression to assess the association between family-adjusted recombination counts and family-adjusted age of mothers at birth and computed the Pearson correlation coefficient, *r*. The family-adjusted value is the difference between the value for a child and the value averaging over all children from a given mother. The family-adjusted values are used to evaluate the effect of age on recombination across transmissions within families, so that detected effects are not confounded by differences among mothers. To examine whether maternal age is the critical variable, as opposed to time between births, we used non-adjusted values to evaluate the maternal age effect across all transmissions with a linear mixed model that allows for correlated recombination rates by including random effects shared within each family. The number of children was also added as a covariate in the model, to adjust for this potential confounder. The results were confirmed by a non-parametric test: we found a significant Spearman correlation for adjusted counts and ages (ρ = −0.25, *p* = 0.0078) and for the non-adjusted values (ρ = −0.31, *p* = 6×10^−4^). To describe the local structure of the relationship between recombination and maternal age, we used a semi-parametric regression model that achieves smoothing using splines and provides a good fit to the data as we move across the range of maternal ages (see Text S1). The R packages lmeSplines and nlme were used to implement our model. The knots were specified at each distinct value of maternal age at birth (*k* = 23) and the smoothing parameter λ was estimated by REML (λ = 22.29). *P*-values were determined based on 10 000 randomized data sets, generated by permuting the maternal age across transmissions. For analyses involving family-adjusted values, permutations were performed within families.

### Chromosome-specific effects

To evaluate chromosome-specific effects, we grouped the transmissions into two categories according to the age of the mother at birth: under 30 years old and 30 years old and above. We tested whether the shift in mean between the two age groups was significant in individual chromosomes (Table S4). Putting aside the significant chromosomes, we used a sign test to evaluate if a systematic effect remained among the non-significant chromosomes, with a standard binomial test used to assess significance. To determine the number of chromosomes expected to show a significant shift given the genome-wide correlation, we performed simulations to redistribute crossovers of each mother randomly across chromosomes, while taking into account the mean number of recombination occuring on each chromosome (see Text S1). We also assessed by simulations whether the shift found in significant chromosomes was significantly different from the shift found in other chromosomes using normalized recombination counts (see Text S1). Normalized values are obtained by dividing the recombination counts by the mean number of recombinations observed on each chromosome across the cohort. *P*-values were obtained using the randomization scheme as described in the previous section.

### Distance from centromere

Centromere positions were extracted from the UCSC Table Browser http://genome.ucsc.edu/cgi-bin/hgText (assembly Mar. 2006). Genomic positions of recombination events were converted to relative positions with respect to centromere location, i.e. a value of 0 for an event at the centromere and 1.0 for an event at chromosomal edges (telomeric regions). Recombination events were grouped in distance bins of 0.05 (Figure 3 and Figure S2b) and 0.1 (Table S3) and were separated according to parental origin and age group (under or over 30 years old). We evaluated the correlation between distances and the number of recombinations inferred in 0.05-bins. The distances were positively correlated with recombination, resulting in a Pearson *r* = 0.86 (*p*<10^−4^) when both paternal and maternal recombinations were considered. The positive correlation remained significant when paternal events (Pearson *r* = 0.79, *p*<10^−4^) and maternal events (Pearson *r* = 0.58, *p* = 0.0047) were considered separately, even though the correlation was weaker in females. *P*-values were determined based on 10 000 permutations of the recombination counts within bins. For each separate bin of size 0.1 and 0.05, we tested whether the shift in mean between mothers younger and older than 30 was significant in individual bins (Figure 3, Figure S2b and Table S3). We assessed by simulations whether the shift found in significant bins was significantly different from the shift found in other bins using normalized recombination counts (see Text S1). We also evaluated the correlation between maternal age and recombination rates using a linear regression model with family-adjusted values for distance bins of 0.1 (Table S3). Similar effects and distributions of events were observed when chromosomal arms shorter and longer than 85 Mb were considered separately.

### Maternal age effect and clinical phenotype

We tested for associations between the LS-CHD phenotype (affected vs. unaffected) and maternal age at birth by an analysis of variance using ANOVA and Kruskal-Wallis rank sum test. The same analyses were performed to test for a relationshp between the clinical phenotype and the number of recombination events found in every child. No significant differences in either recombination rates or maternal age at birth were observed between unaffected and affected individuals.

### Factors influencing power to detect the maternal age effect

To evaluate the effect of sampling on the correlation between recombination rates and maternal age, we used resampling methods. We performed boostrap analyses over families within both the French-Canadian and Hutterite datasets. We also used a jackknife approach to generate samples similar to the French-Canadian dataset, using subsets of available and simulated data (Text S1).

Power to detect variation among transmissions can be affected by low SNP density. To evaluate the impact of different SNP density on our results, we used the –thin option of PLINK toolset [56] to keep only a random 80%, 40%, 30%, 20% and 5% of SNPs. Five percent of SNPs corresponds to analysis with an average of 1000 informative markers per mother, which is the marker density used in the Icelandic study [16]. Four reduced datasets were created per SNP density. Recombinations were inferred for the 20 reduced datasets and the maternal age effect was evaluated on family-adjusted values.

Using approximations for the ages of individuals can lower the power to detect a correlation between maternal age at birth and recombination. Ages of all individuals (children and parents) were rounded up to the nearest five years and maternal age at birth was calculated by substracting the new child's age from the new mother's age. Since linear relationship between recombination rate and maternal age is no longer consistent with this data, the maternal age effect was evaluated by ANOVA, categorising estimates based on approximate ages.

### Analyses of the maternal age effect on recombination found in Hutterites

We were provided access to the list of recombination events inferred in the Hutterite study and parental age at birth for individuals in 52 nuclear families, providing information for 282 female meiosis out of 364 analysed by Coop and colleagues [11]. We evaluated the genome-wide correlation between family-adjusted recombination counts and maternal age using Pearson and Spearman correlation coefficients. In large families (>3 children), events that are not unique within a family, for example, seen in at least two children, were called by Coop et al. [11]. All analyses were performed with the unique events only (947 events were removed) and the results remained unchanged.

Effects specific to chromosomal regions and chromosomes were evaluated as previously described. To obtain the probability, by chance alone, of at least two chromosomes showing a significant decay, we assume that the shift has the same probability to be either positive or negative. Using a binomial distribution, we computed the probability of having at least *k* = 2 chromosomes out of *n* = 22 at *p* = 0.0355, *p_chr_* = (1−P(*k* = 0)−P(*k* = 1))·0.5^2^∼0.182·0.25∼0.0455. We also performed simulations where we redistributed, for each transmission, the recombination events uniformly across chromosomes (Text S1) and computed the shift and significance by chromosome. We find that, among 5000 simulations, only 201 had at least 2 chromosomes exhibiting a negative shift with a *p*-value lower than 0.036 (*p_chr_* = 0.0402).

To compare the results observed in the French-Canadian and Hutterite cohorts with those obtained by Kong and colleagues [16], we treated maternal age as a categorical variable. For each cohort, transmissions were grouped into 4 categories according to age of the mother at birth: under 25 years old, between 25–29 years old, between 30 to 34 years old, 35 years old and above. We tested differences among categories by ANOVA (French-Canadians: *p* = 5·10^−4^, Hutterites: *p* = 0.091) and using Kruskal-Wallis rank sum test (French-Canadians: χ^2^ = 10.77 *p* = 0.013, Hutterites: χ^2^ = 6.32 *p* = 0.098). All *p*-values were obtained using the randomization scheme described above.

## Supporting Information

Dataset S1 Genotypic data of the 478 French-Canadian individuals after stringent quality control. The files were prepared using Plink v1.09 software [56] in the binary file format (BED/BIM/FAM).
(BZ2)

Figure S1 Congruence of Phase II Hapmap recombination hotspots with events localized between markers less than 30 KB apart. Positions of active hotspots on each autosome in mothers under 30 years old (blue) and 30 years old and over (red) are plotted. Black lines represent positions of centromeres.
(TIFF)

Figure S2 Maternal age effect in the Hutterites study. (a) Scatterplot and fitted regression functions showing negative correlation between the maternal age at birth and the number of recombination events in offspring. The red dashed line represents the linear regression (β = 0.17, *p* = 0.031, r^2^ = 0.012) and the solid blue line represents the result of the linear spline regression with knots at each distinct value of maternal age at birth (λ = 17.05, *p* = 0.0354, r^2^ = 0.012). (b) Distribution of recombination events along chromosomal arms (see Figure 3 for detailed description). Significance of the shift at the 5% level (*) is assessed by permutations.
(TIFF)

Figure S3 Relationship between maternal age and recombination in autosomes using categorized data for (a) the French-Canadian cohort and (b) the Hutterite cohort. The number of recombinations for all transmissions are plotted (smaller dots), sample means and standard errors for each age group are shown. The numbers of transmissions (n) in each category are reported.
(TIFF)

Table S1 Significant variation among autosomes in number of recombination events among male and female transmissions. The mixed and adjusted models are described in [11] and significance was assessed based on permutations (see Materials and Methods) using a likelihood-ratio test. Significant p-values (*p*<0.05) are reported, otherwise, they are not significant (n.s.) is indicated. Values for chromosomes that were significant among the Hutterites (see Table S1 in [11]) are presented in red.
(PDF)

Table S2 Exclusion of double recombinants. Correlation between family-adjusted age of mothers at birth and family-adjusted recombination counts was evaluated without double recombinants occurring between 2, 5, 10 or 20 Mb intervals. Permutations were used to assess significance.
(PDF)

Table S3 Correlations between recombination counts and maternal age along chromosomal arms. Shifts of the mean number of maternal crossovers between mothers under and over 30 years of age are presented at different distances relative to centromere position. Linear correlations are evaluated using family-adjusted values grouped in 10 bins of distance relative to centromere location. Permutations were used to assess significance (*p*<0.05) and significant results are reported in bold.
(PDF)

Table S4 Mean number of recombination events among maternal transmissions for each autosome in the French-Canadian and Hutterite studies. For each study, transmissions are partitioned according to the age of the mother at birth (mothers of 30 years-old are part of the over-30 group). Permutations were used to test whether the shift was significant and significant results are reported in bold.
(PDF)

Text S1 Supplementary methods.
(DOC)
