# Supplementary figures and images for: Age-Dependent Recombination Rates in Human Pedigrees
Source: PLoS Genet. 2011 Sep 1;7(9):e1002251. doi: 10.1371/journal.pgen.1002251 (PMC3164683; doi:10.1371/journal.pgen.1002251)

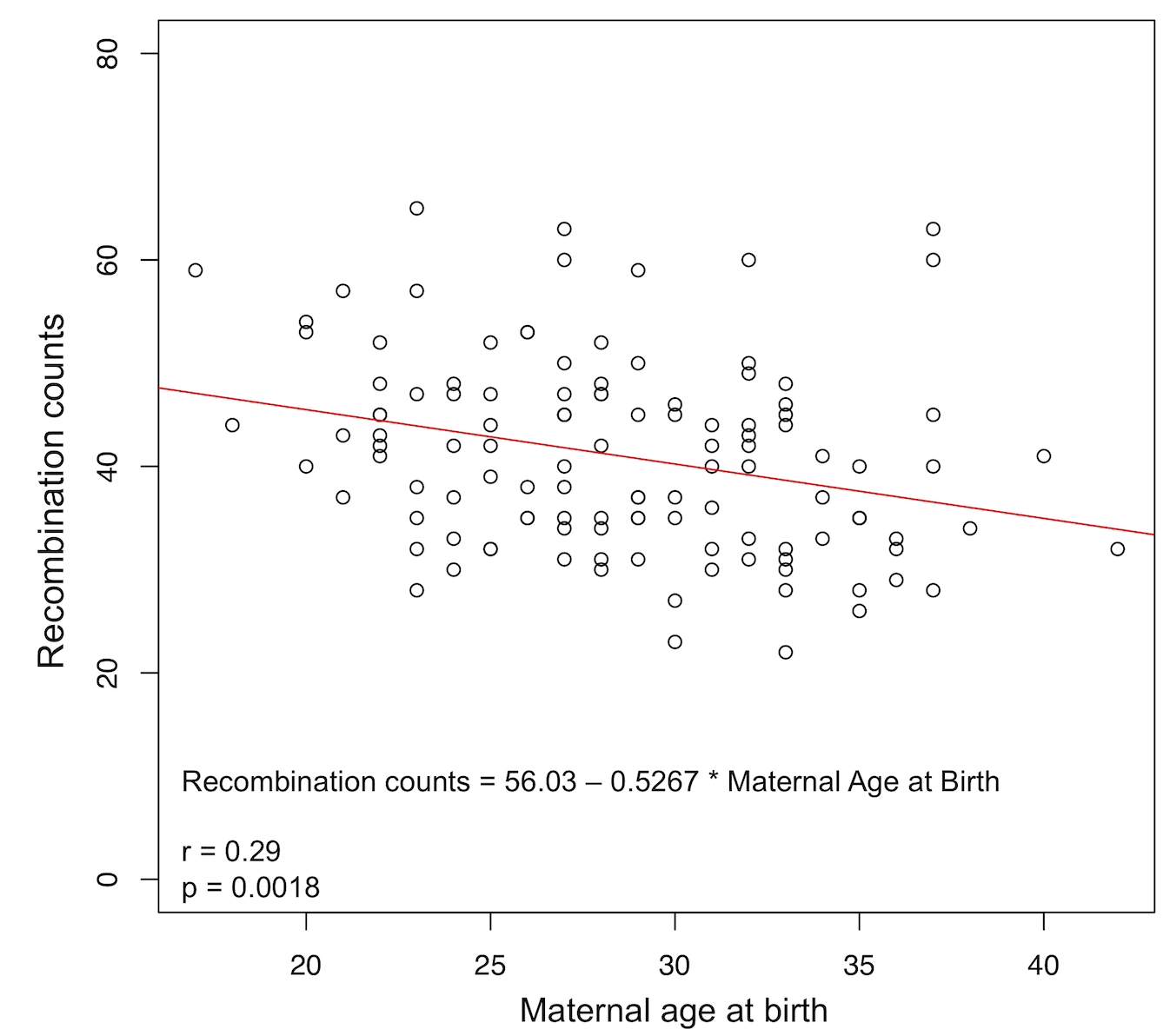

Supplement: Figure S1 — Congruence of Phase II Hapmap recombination hotspots with events localized between markers less than 30 KB apart. Positions of active hotspots on each autosome in mothers under 30 years old (blue) and 30 years old and over (red) are plotted. Black lines represent positions of centromeres. (TIFF) [file pgen.1002251.s002.tiff]

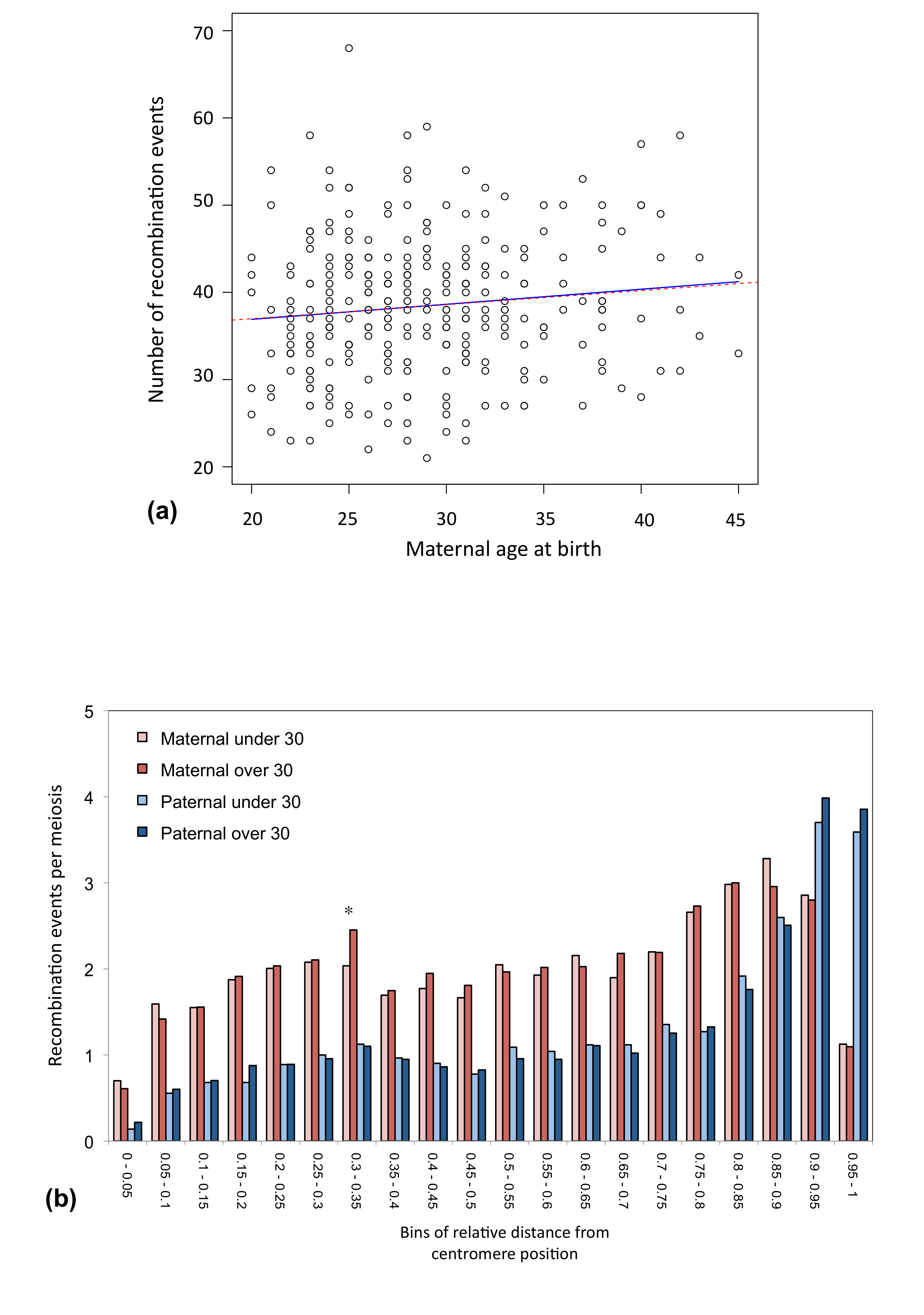

Supplement: Figure S2 — Maternal age effect in the Hutterites study. (a) Scatterplot and fitted regression functions showing negative correlation between the maternal age at birth and the number of recombination events in offspring. The red dashed line represents the linear regression (β = 0.17, p = 0.031, r2 = 0.012) and the solid blue line represents the result of the linear spline regression with knots at each distinct value of maternal age at birth (λ = 17.05, p = 0.0354, r2 = 0.012). (b) Distribution of recombination events along chromosomal arms (see Figure 3 for detailed description). Significance of the shift at the 5% level (*) is assessed by permutations. (TIFF) [file pgen.1002251.s003.tiff]

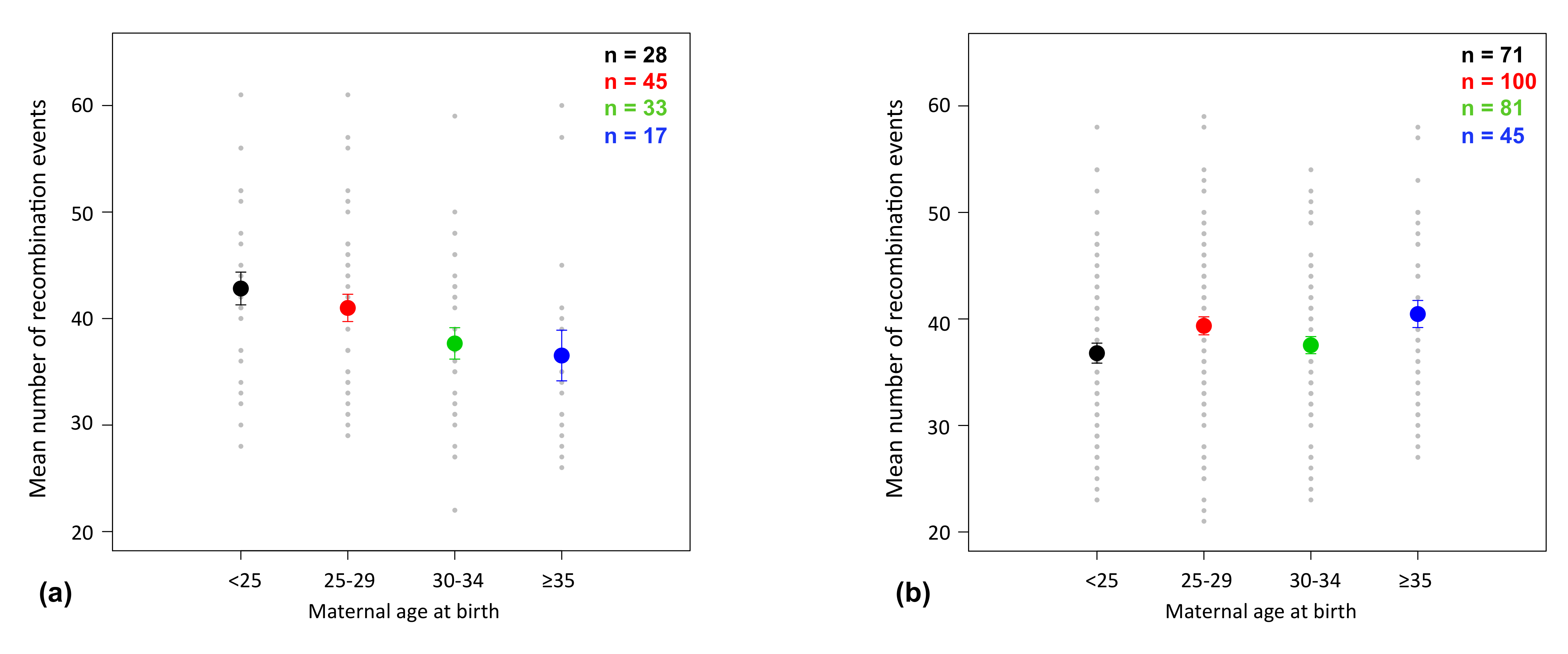

Supplement: Figure S3 — Relationship between maternal age and recombination in autosomes using categorized data for (a) the French-Canadian cohort and (b) the Hutterite cohort. The number of recombinations for all transmissions are plotted (smaller dots), sample means and standard errors for each age group are shown. The numbers of transmissions (n) in each category are reported. (TIFF) [file pgen.1002251.s004.tiff]
