# Supplementary material for: Age-Dependent Recombination Rates in Human Pedigrees
Source: PLoS Genet. 2011 Sep 1;7(9):e1002251. doi: 10.1371/journal.pgen.1002251 (PMC3164683; doi:10.1371/journal.pgen.1002251)
Supplement: Table S1 — Significant variation among autosomes in number of recombination events among male and female transmissions. The mixed and adjusted models are described in [11] and significance was assessed based on permutations (see Materials and Methods) using a likelihood-ratio test. Significant p-values (p<0.05) are reported, otherwise, they are not significant (n.s.) is indicated. Values for chromosomes that were significant among the Hutterites (see Table S1 in [11]) are presented in red. (PDF) [file pgen.1002251.s005.pdf]

Chr	Males p-value		Females p-value	
	Mixed model	Adjusted model	Mixed model	Adjusted model
1	ns	ns	ns	ns
2	ns	ns	ns	ns
3	ns	ns	** **0.005648**	ns
4	ns	ns	ns	ns
5	* **0.0476**	ns	** **0.006067**	ns
6	ns	ns	** **0.002188**	ns
7	ns	ns	ns	ns
8	ns	ns	** **0.000868**	ns
9	ns	ns	ns	ns
10	ns	ns	ns	ns
11	ns	ns	ns	ns
12	* **0.0452**	* **0.0400**	** **0.000738**	ns
13	** **0.00149**	** **0.00582**	ns	ns
14	ns	ns	ns	ns
15	ns	ns	** **0.00114**	ns
16	** **0.00872**	ns	** **0.00391**	* **0.0262**
17	* **0.0189**	ns	ns	ns
18	ns	ns	ns	ns
19	** **0.00750**	* **0.0294**	* **0.0251**	ns
20	ns	ns	* **0.04965**	ns
21	ns	ns	* **0.02196**	* **0.0272**
22	ns	ns	ns	ns
