# Supplementary material for: Age-Dependent Recombination Rates in Human Pedigrees
Source: PLoS Genet. 2011 Sep 1;7(9):e1002251. doi: 10.1371/journal.pgen.1002251 (PMC3164683; doi:10.1371/journal.pgen.1002251)
Supplement: Table S2 — Exclusion of double recombinants. Correlation between family-adjusted age of mothers at birth and family-adjusted recombination counts was evaluated without double recombinants occurring between 2, 5, 10 or 20 Mb intervals. Permutations were used to assess significance. (PDF) [file pgen.1002251.s006.pdf]

Exclusion interval	β	*P*-value	Pearson *r*
< 2 Mb	-0.42	0.0012	-0.225
< 5 Mb	-0.44	0.0013	-0.214
< 10 Mb	-0.42	0.0018	-0.207
< 20 Mb	-0.42	0.0056	-0.239
