# Supplementary material for: Age-Dependent Recombination Rates in Human Pedigrees
Source: PLoS Genet. 2011 Sep 1;7(9):e1002251. doi: 10.1371/journal.pgen.1002251 (PMC3164683; doi:10.1371/journal.pgen.1002251)
Supplement: Table S3 — Correlations between recombination counts and maternal age along chromosomal arms. Shifts of the mean number of maternal crossovers between mothers under and over 30 years of age are presented at different distances relative to centromere position. Linear correlations are evaluated using family-adjusted values grouped in 10 bins of distance relative to centromere location. Permutations were used to assess significance (p<0.05) and significant results are reported in bold. (PDF) [file pgen.1002251.s007.pdf]

Bins		Shift in recombination counts between mothers under and over 30			Linear correlation using adjusted values		
min	max	Shift	Direction	Shift *p*-value	β	Pearson r	*p*-value
0	0.1	0.30	-	0.325	0.03	0.068	0.512
0.1	0.2	0.04	+	0.911	0.02	0.046	0.652
0.2	0.3	0.06	+	0.871	- 0.04	- 0.093	0.366
0.3	0.4	1.07	-	**0.006**	- 0.12	- 0.269	**0.007**
0.4	0.5	1.05	-	**0.007**	- 0.06	- 0.139	0.18
0.5	0.6	1.06	-	**0.005**	- 0.04	- 0.091	0.375
0.6	0.7	0.01	+	0.984	0.004	0.009	0.927
0.7	0.8	0.99	-	**0.01**	- 0.13	- 0.268	**0.007**
0.8	0.9	1.39	-	**0.003**	- 0.16	- 0.247	**0.014**
0.9	0.0	0.37	-	0.479	- 0.09	- 0.149	0.146
