# Supplementary material for: Age-Dependent Recombination Rates in Human Pedigrees
Source: PLoS Genet. 2011 Sep 1;7(9):e1002251. doi: 10.1371/journal.pgen.1002251 (PMC3164683; doi:10.1371/journal.pgen.1002251)
Supplement: Table S4 — Mean number of recombination events among maternal transmissions for each autosome in the French-Canadian and Hutterite studies. For each study, transmissions are partitioned according to the age of the mother at birth (mothers of 30 years-old are part of the over-30 group). Permutations were used to test whether the shift was significant and significant results are reported in bold. (PDF) [file pgen.1002251.s008.pdf]

Chromosomes	Means in the French-Canadian cohort			Permutation test *p*-values	Means in the Hutterite cohort			Permutation test *p*-values
	Mother under 30	Mother over 30	Sign		Mother under 30	Mother over 30	Sign	
Chr 1	3.19	2.90	-	0.1843	3.29	3.15	-	0.4335
Chr 2	3.18	2.92	-	0.1829	2.88	3.12	+	0.1869
Chr 3	2.47	2.31	-	0.2641	2.46	2.71	+	0.1072
Chr 4	2.42	2.17	-	0.1585	2.39	2.60	+	0.1975
Chr 5	2.58	2.15	-	**0.0302**	2.33	2.30	-	0.8127
Chr 6	2.71	2.00	-	**0.0027**	2.32	2.37	+	0.7512
Chr 7	2.35	1.96	-	**0.0302**	2.09	2.25	+	0.2542
Chr 8	2.32	1.75	-	**0.0131**	2.07	2.07	+	0.9792
Chr 9	2.00	1.60	-	**0.0421**	1.90	1.99	+	0.5435
Chr 10	2.21	1.73	-	**0.0163**	2.26	2.02	-	0.0735
Chr 11	1.81	1.73	-	0.3621	1.81	1.76	-	0.6742
Chr 12	1.94	1.87	-	0.3746	1.92	2.00	+	0.5317
Chr 13	1.55	1.48	-	0.3574	1.45	1.42	-	0.7540
Chr 14	1.29	1.19	-	0.2981	1.34	1.49	+	0.1628
Chr 15	1.65	1.02	-	**0.0005**	1.39	1.36	-	0.7823
Chr 16	1.74	1.58	-	0.2267	1.60	1.54	-	0.5923
Chr 17	1.48	1.46	-	0.4469	1.58	1.43	-	0.2075
Chr 18	1.58	1.25	-	**0.0338**	1.39	1.47	+	0.5086
Chr 19	1.19	0.98	-	0.1007	1.07	1.02	-	0.5686
Chr 20	1.37	1.08	-	**0.0267**	1.37	1.14	-	**0.0354**
Chr 21	0.81	0.71	-	0.2303	0.66	0.59	-	0.4006
Chr 22	0.87	0.73	-	0.1556	0.77	0.60	-	**0.0321**
Autosomes	43.07	38.04	-	**0.0011**	40.10	40.63	+	0.5801
