# Supplementary material for: Age-Dependent Recombination Rates in Human Pedigrees
Source: PLoS Genet. 2011 Sep 1;7(9):e1002251. doi: 10.1371/journal.pgen.1002251 (PMC3164683; doi:10.1371/journal.pgen.1002251)
Supplement: Text S1 — Supplementary methods. (DOC) [file pgen.1002251.s009.doc]

**Text S1**

For **Age-dependent recombination rates in human pedigrees.**

Julie Hussin^1,2^, Marie-Helene Roy-Gagnon^2,3^, Roxanne Gendron^2^, Gregor Andelfinger^2,4^ and Philip Awadalla^2,4^

^1^Department of Biochemistry, Faculty of Medicine, University of Montreal, QC, Canada ; ^2^Ste-Justine Hospital Research Centre, Montreal, QC, Canada ; ^3^Department of Social and Preventive Medicine, Faculty of Medicine, University of Montreal, QC, Canada ; ^4^Department of Pediatrics, Faculty of Medicine, University of Montreal, QC, Canada.

**Supplementary Methods**

**1. Spline smoothing with a linear mixed model**

We used a linear mixed model to implement a semi-parametric regression model of the relationship between recombination counts and maternal age at birth for nuclear families.

We seek to fit the following model :

rec.counts = *m(*age.at.birth*)* + *h* + ε

where *m* is the smoothing function of ages at birth, with the random effects *h*, with variance , capturing the within family correlation structure and ε representing an uncorrelated random error term, with variance .

We used the R packages lmeSplines and nlme to implement our model. The package lmeSplines adds smoothing spline modelling capability to mixed linear models. We located a knot at each distinct value of age, although choosing a smaller number of knots did not change the results. The smoothing parameter is estimated by maximizing the restricted log-likelihood.

This model was used to analyse the correlation between recombination counts and maternal age at birth in the French-Canadian cohort and in the Hutterite cohort . In the French-Canadians, the spline fit revealed new features of the data (Figure 1) although the improvement in goodness of fit provided by the linear spline model, evaluated by ANOVA, is not significant. In the Hutterites, the spline fit is very close to the linear fit (Figure S2a).

**2. Evaluation of chromosome-specific effects**

To evaluate if the significant correlations found on 9 out of 22 chromosomes are due to chromosome-specific effects and are not simply due to lack of power to detect the correlation on all the chromosomes, we used two simulation-based approaches. The first approach was also used with the data from the Hutterite cohort to evaluate the probability of having 2 chromosomes out of 22 exhibiting a significant negative correlation.

2.1 Evaluation of the expected number of significant chromosomes

We performed 5000 simulations where maternal recombination events inferred in each child were redistributed uniformly across chromosomes, while taking into account the mean number of recombination events expected to occur on each chromosome. Specifically, for each transmission, the number of crossovers for each chromosome was drawn from a multinomial distribution with probabilities *c* and sample size *n*, where *n* is the total number of recombination events for that transmission and *c* = {*c_1_,c_2_,…,c_22_*} with *c_i_* the proportion of events found on chromosome *i* across all transmissions. We grouped the transmissions into two categories according to the age of the mother at birth: under 30 years old and 30 years old and above. For each simulation, we tested whether the shift in mean between the two age groups was significant and evaluated the number of chromosomes exhibiting a significant shift.

For the French-Canadian cohort, the maximum number of significant chromosomes found among the 5000 simulations was 7 and only 28/5000 had more than 4 significant chromosomes. For the Hutterite cohort, only 201 simulations among 5000 showed at least 2 chromosomes exhibiting a negative shift and a *p*-value lower than 0.036 (one tailed *p* = 0.0402).

2.2 Differences between significant and non-significant chromosomes

We separated the 9 significant chromosomes (SC) from the 13 non-significant chromosomes (NSC) found in the French-Canadian cohort. No significant correlation with maternal age was found for NSC (β = -0.13, r = -0.14, *p* = 0.101). We normalized the values by dividing the recombination counts by the average number of recombinations observed for each chromosome across the cohort, we grouped the SC and NSC together and computed the shifts between older and younger mothers for the two groups separately. The normalized shift estimates are 0.251 for SC and 0.082 for NSC. We then permuted the normalized values among chromosomes, and grouped the nine chromosomes having the largest shifts separately from the remaining ones. The shifts between older and younger mothers for the two groups were then calculated. The difference observed in the shifts between SC and NSC, 0.169, is significantly different from that expected based on simulations (*p* = 0.0012). Even though we expect significant chromosomes to show a higher average effect than the non-significant ones, such a significant difference between the two groups was not seen in the simulations above (section 2.1) where the effect is distributed uniformly across chromosomes.

**3. Evaluation of effects in specific chromosomal regions**

Maternal recombination events were divided in bins of relative distance from centromere of 0.1 and were separated according to age group of the mother at time of birth (under or over 30 years old). For each bin, we tested whether the shift in mean between the two groups was significant. The five significant bins are 0.3-0.4, 0.4-0.5, 0.5-0.6, 0.7-0.8 and 0.8-0.9 (Table S3). For each bin, the recombination counts were normalized by dividing each count by the average number of recombination counts observed in the bin across the cohort, and the normalized shifts were computed. The normalized shift estimates are 1.08 for the significant bins and 0.13 for the remaining ones, leading to a difference in shift of 0.95. To assess whether the normalized shift for the significant bins was significantly higher than the shift found in non-significant bins, we permuted the normalized values between bins. We then grouped the five bins showing the largest shifts separately from the remaining ones and computed the difference observed in the shifts between these two groups. The difference in shift observed in the data is significantly higher (*p =*0.0464) than what is expected based on these simulations.

**4. Evaluation of sample size effects**

To evaluate the impact of sample size effects on the results, we used bootstrap and jackknife procedures :

4.1 Bootstrapping over families

We randomly draw families with replacement from the set of 34 French-Canadian families, to create 5000 simulated datasets and we examined the correlation between family-adjusted recombination counts and family-adjusted maternal ages at birth. For 4051/5000 bootstrap repetitions (81%), we obtain a significant negative correlation. Only 2/5000 (0.04%) exhibit a positive β, but the correlations were not significant. We performed the same experiment with the 52 families from the Hutterite cohort. For 3200/5000 bootstrap repetitions (64%), we obtain a significant positive correlation and 107/5000 (2.1%) exhibit a negative β, but none of them were significant. These results show that the confidence intervals for the correlation coefficients observed in the French-Canadian (95% CI -0.84 to -0.18) and Hutterite cohort (95% CI -0.10 to 0.43) do not deviate considerably from the negative and positive point estimates, respectively.

4.2 Jackknifing over Hutterites families to match the French-Canadian cohort

We used the 52 families from the Hutterite cohort to create samples of 34 families with the same number of children as in the French-Canadian families. Only 5/5000 simulations showed a significant negative correlation between family-adjusted values. This analysis suggests that it is unlikely (one-tailed *p* = 0.001) to observe a negative maternal age effect in a subset of 34 out of 52 families exhibiting a positive maternal age effect of ~0.22 recombinations per year.

4.3 Simulation of the Icelandic cohort and jackknifing.

We used the informations provided by Kong and colleagues to simulate a dataset, with the same characteristics :

- the total number of nuclear families with at least 3 children (2177) ;
- the number of sampled children per family (Table 1 in ) ;
- the distribution of mother’s age at birth (Table 2 in ) ;
- the average number of recombination events per maternal meiosis of 44.6, reported in , with standard deviation of σ = 8 recombinations (σ_Hutterites_ = 7.90 and σ_French-Canadians_ = 8.28) ;
- the estimated maternal age effect of 0.043 recombinations per year.

We then used this simulated dataset to create samples of 34 families with the same number of children as in our French-Canadian families. Only 94/5000 simulations showed a significant negative correlation between family-adjusted values. This analysis suggests that it is unlikely (one-tailed *p* = 0.0188) to observe a negative maternal age effect in a subset of 34 out of 2177 families exhibiting a positive maternal age effect of ~0.045 recombinations per year.

**5. MODEL: Recombination provides a reduced protection against non-disjunction as women age.**

We propose that a high recombination rate protects an oocyte ovulated earlier in life from non-disjunction, but that this protection becomes less efficient with increasing maternal age.

Let be the probability of proper disjunction in an oocyte with r crossovers after k years. Under our hypothesis, decreases with k for r ≥ ε (with ε ≥ 0).

Let *R_k_* be the number of crossovers observed in a properly disjoined oocyte after k years. The mean number of crossovers in these normal oocyte (*E_N_*) is :

with the probability of finding r recombinations in an oocytes, assuming here that this probability does not depends on k.

For therefore (see Figure 5).

Under this model, the mean number of crossovers in properly disjoined oocytes (*E_N_*) is expected to decrease with maternal age, whereas the mean number of recombination in non-disjoined oocytes (*E_A_*) is expected to increase with maternal age.

**REFERENCES**

1. Gurrin LC, Scurrah KJ, Hazelton ML (2005) Tutorial in biostatistics: spline smoothing with linear mixed models. Stat Med 24: 3361-3381.

2. Coop G, Wen X, Ober C, Pritchard JK, Przeworski M (2008) High-resolution mapping of crossovers reveals extensive variation in fine-scale recombination patterns among humans. Science 319: 1395-1398.

3. Kong A, Barnard J, Gudbjartsson DF, Thorleifsson G, Jonsdottir G, et al. (2004) Recombination rate and reproductive success in humans. Nat Genet 36: 1203-1206.

4. Kong A, Gudbjartsson DF, Sainz J, Jonsdottir GM, Gudjonsson SA, et al. (2002) A high-resolution recombination map of the human genome. Nat Genet 31: 241-247.
